# TAp63, a methotrexate target in CD4^+^ T cells, suppresses Foxp3 expression and exacerbates autoimmune arthritis

**DOI:** 10.1172/jci.insight.164778

**Published:** 2023-05-22

**Authors:** Kensuke Suga, Akira Suto, Shigeru Tanaka, Yutaka Sugawara, Takahiro Kageyama, Junichi Ishikawa, Yoshie Sanayama, Kei Ikeda, Shunsuke Furuta, Shin-Ichiro Kagami, Arifumi Iwata, Koichi Hirose, Kotaro Suzuki, Osamu Ohara, Hiroshi Nakajima

**Affiliations:** 1 Department of Allergy and Clinical Immunology, Graduate School of Medicine, and; 2 Institute for Advanced Academic Research, Chiba University, Chiba, Japan.; 3 Research Center for Allergy and Clinical Immunology, Asahi General Hospital, Asahi, Chiba, Japan.; 4 Department of Applied Genomics, Kazusa DNA Research Institute, Kisarazu, Chiba, Japan.

**Keywords:** Autoimmunity, Immunology, Rheumatology, T cells

## Abstract

Methotrexate (MTX) is a standard, first-line therapy for rheumatoid arthritis (RA); however, its precise mechanisms of action other than antifolate activity are largely unknown. We performed DNA microarray analyses of CD4^+^ T cells in patients with RA before and after MTX treatment and found that *TP63* was the most significantly downregulated gene after MTX treatment. TAp63, an isoform of TP63, was highly expressed in human IL-17–producing Th (Th17) cells and was suppressed by MTX in vitro. Murine TAp63 was expressed at high levels in Th cells and at lower levels in thymus-derived Treg cells. Importantly, TAp63 knockdown in murine Th17 cells ameliorated the adoptive transfer arthritis model. RNA-Seq analyses of human Th17 cells overexpressing TAp63 and those with TAp63 knockdown identified *FOXP3* as a possible TAp63 target gene. TAp63 knockdown in CD4^+^ T cells cultured under Th17 conditions with low-dose IL-6 increased Foxp3 expression, suggesting that TAp63 balances Th17 cells and Treg cells. Mechanistically, TAp63 knockdown in murine induced Treg (iTreg) cells promoted hypomethylation of conserved noncoding sequence 2 (CNS2) of the Foxp3 gene and enhanced the suppressive function of iTreg cells. Reporter analyses revealed that TAp63 suppressed the activation of the Foxp3 CNS2 enhancer. Collectively, TAp63 suppresses Foxp3 expression and exacerbates autoimmune arthritis.

## Graphical abstract

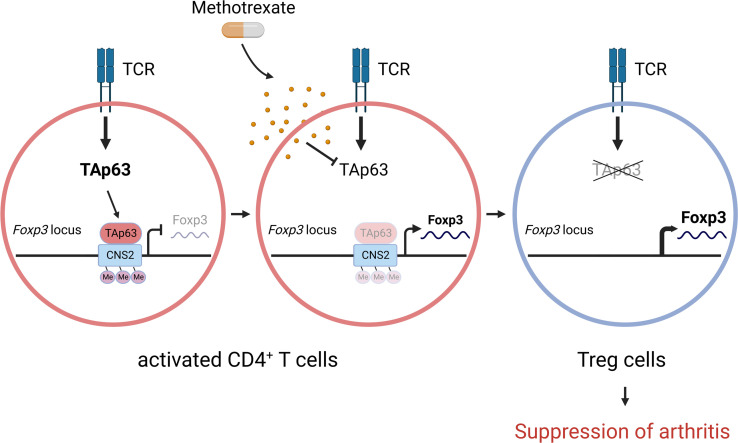

## Introduction

Rheumatoid arthritis (RA) is a systemic and heterogeneous autoimmune disease caused by impaired self-tolerance and characterized by chronic and destructive joint disease, leading to functional disability and premature mortality (1). Early diagnosis, early intervention, and tight control with potent antirheumatic drugs have improved clinical, structural, and functional outcomes of patients with RA in the past 20 years (2–4), with methotrexate (MTX) playing a central role in such a therapeutic strategy (5). MTX is a folic acid antagonist that competes with dihydrofolate reductase, resulting in decreased purine and pyrimidine biosynthesis and effectively disturbing DNA replication and cell proliferation (6, 7). Indeed, folate metabolism in peripheral blood cells is markedly upregulated in patients with active RA, and the treatment with MTX restores folate metabolism to normal levels (8). Other than the inhibition of folate metabolism, it has been shown that methotrexate polyglutamate, a steady state of intracellular MTX, inhibits 5-aminoimidazole-4-carboxamide ribonucleotide (AICAR) formyltransferase (ATIC), leads to intracellular AICAR accumulation, and promotes adenosine release, which diminishes inflammation (7).

The presence of autoantibodies such as rheumatoid factor and anti-citrullinated protein antibody suggests that adaptive immunity is involved in the pathogenesis of RA (9). Indeed, GWAS have shown that the MHC class II molecule HLA-DRB1 is the most significant susceptibility locus in RA (10, 11), suggesting an essential role of CD4^+^ T cells in the pathogenesis of RA. In this regard, the balance between the activation of IL-17–producing Th (Th17) cells and CD4^+^ Treg cells is believed to be important (12). Transcription factor Foxp3 is indispensable for the development and maintenance of Treg cells, and demethylation of conserved noncoding sequence 2 (CNS2) of Foxp3 locus is required for stable expression of Foxp3 in Treg cells (13). In addition, it has recently been shown that autoimmune-associated SNPs are highly enriched in Treg cell–specific superenhancers and demethylated regions (14), suggesting that the maintenance failure of Treg cells could be involved in the pathogenesis of RA. Given the effectiveness of MTX in patients with RA, it is plausible that MTX targets CD4^+^ T cells to exert its clinical effectiveness. Although it has been reported that MTX suppresses T cell activity and proliferation (15–18) and increases Treg cells (19, 20), the mechanisms of action of MTX on T cells have poorly been understood.

This study aimed to identify candidate genes regulated by MTX treatment in CD4^+^ T cells in patients with RA. We identified a candidate molecule, TAp63, an isoform of TP63, by analyzing comprehensive gene expression profiles of CD4^+^ T cells of patients with RA before and after MTX treatment.

## Results

### mRNA expression of TAp63 in CD4^+^ T cells decreases after MTX treatment in patients with RA.

**Figure 1:**
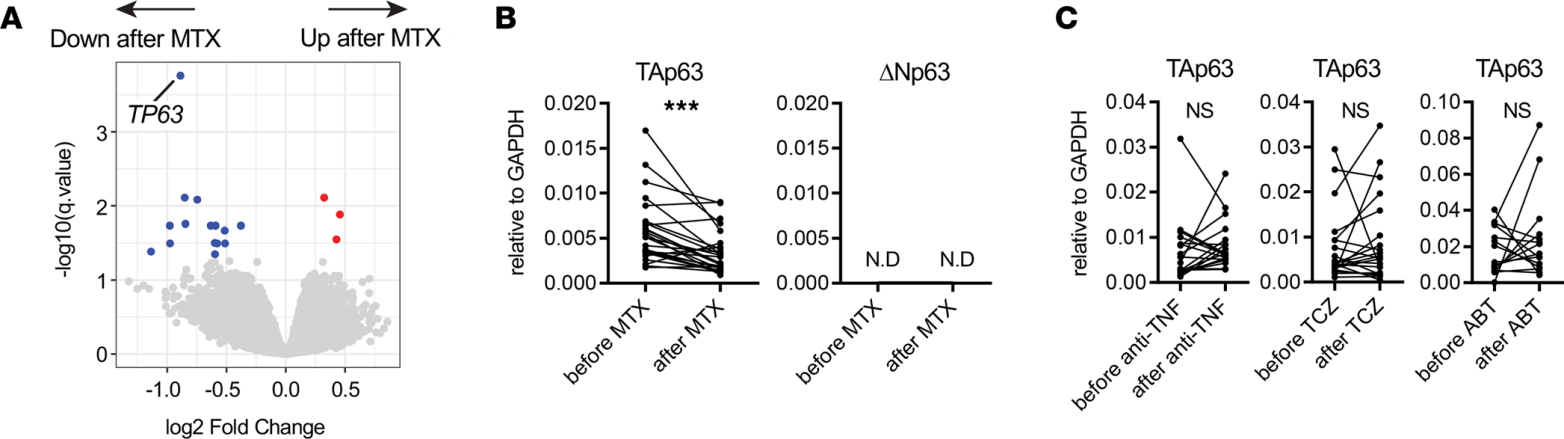
Expression levels of TP63 isoforms in CD4^+^ T cells before and after treatment. (**A**) Volcano plot of transcriptome of CD4^+^ T cells from patients with RA before and after MTX treatment. DEGs (*q* < 0.05 and *P* < 0.05) are highlighted in red (upregulated after MTX) or blue (downregulated after MTX). (**B**) mRNA levels of TAp63 and ΔNp63 in peripheral CD4^+^ T cells before and after MTX treatment were determined by qPCR analysis. *n* = 28. ***P < 0.0001 (Wilcoxon matched-pairs signed-rank test). ND, not detected. (**C**) mRNA levels of TAp63 in peripheral CD4^+^ T cells before and after treatment with biologics were determined by qPCR analysis. Samples from patients treated with TNF antagonists (*n* = 19), TCZ (*n* = 21), or ABT (*n* = 15) were analyzed. Wilcoxon matched-pairs signed-rank test.

To identify genes whose expression in CD4^+^ T cells is regulated by MTX, we examined comprehensive gene expression profiles of CD4^+^ T cells of patients with RA before and after MTX treatment, using DNA microarray analysis. We identified several differentially expressed genes (DEGs), as described previously (21). In this study, we focused on *TP63* because it showed a maximum reduction in signal intensity following MTX treatment (Figure 1A and Table 1). Although not statistically significant, there is a weak correlation between the change in disease activity estimated by Disease Activity Score 28 erythrocyte sedimentation rate (DAS28-ESR) and the change of TAp63 expression in patients with RA treated with MTX (ρ = 0.2886; *P* = 0.1715) (Supplemental Figure 1; supplemental material available online with this article; https://doi.org/10.1172/jci.insight.164778DS1).

TP63 has 2 groups of isoforms with distinct transcription start sites: TAp63 and Δ N isoform (ΔNp63), which lacks the N-terminal region of TAp63 (22). To determine which isoform of TP63 is preferentially expressed in CD4^+^ T cells in patients with RA, real-time PCR (qPCR) analysis was performed on the same samples subjected to the DNA microarray analysis. The expression of TAp63 isoform but not ΔNp63 isoform was detected in CD4^+^ T cells in patients with RA (Figure 1B). Consistent with microarray analyses, the relative expression of TAp63 isoform significantly decreased after MTX treatment in patients with RA (Figure 1B).

To determine whether the decrease in TAp63 expression in CD4^+^ T cells is specific to MTX treatment, we performed qPCR analyses on CD4^+^ T cells of patients with RA treated with biologics (Table 2). The expression levels of TAp63 did not change significantly after treatment with TNF antagonists, tocilizumab (TCZ), or abatacept (ABT) (*P* = 0.26, 0.47, and 0.56, respectively) (Figure 1C). These results suggest that the decrease in TAp63 expression in CD4^+^ T cells is associated with MTX treatment.

### TAp63 is expressed in human and murine CD4^+^ T cells and suppressed by MTX.

**Figure 2:**
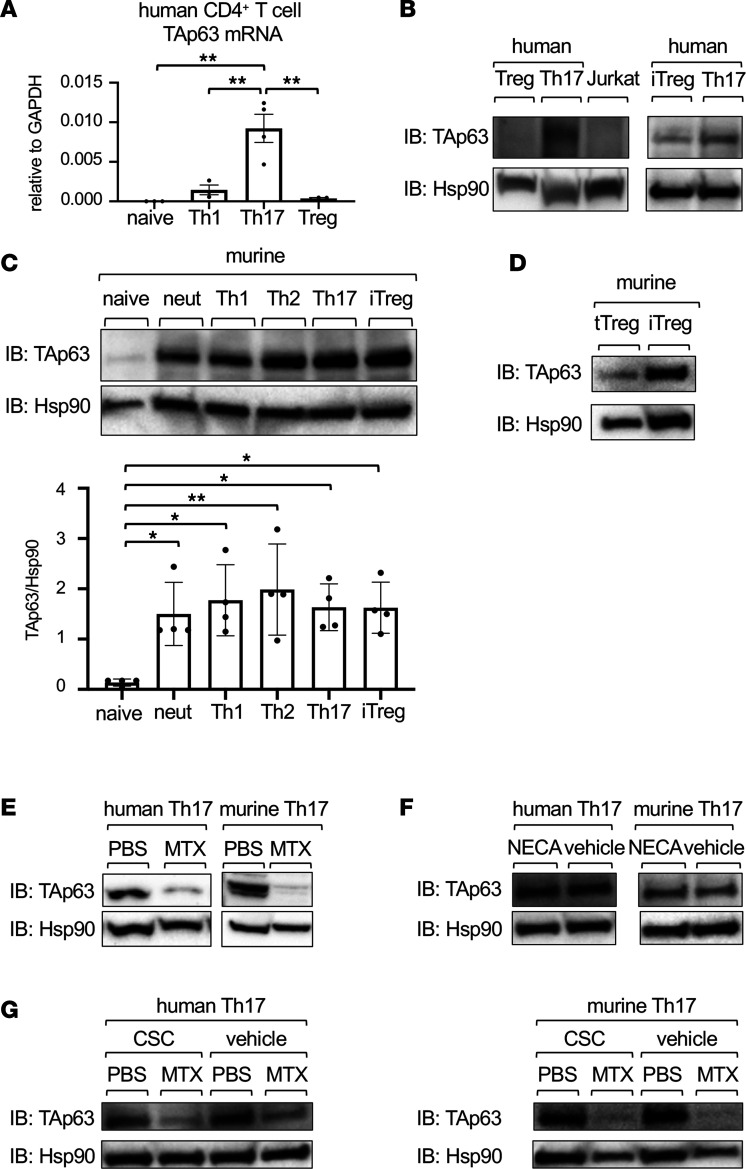
Expression levels of TAp63 in CD4^+^ T cell subpopulations. (**A**) mRNA levels of TAp63 in naive CD4^+^ T cells, Th1 cells, Th17 cells, and Treg cells of healthy individuals were determined by qPCR analysis. Data are reported as mean ± SEM. *n* = 3–4. **P < 0.01 by 1-way ANOVA followed by Tukey’s test. (**B**) Representative Western blot analysis of TAp63 and Hsp90 in human Treg cells, Th17 cells, and Jurkat cells (left panels), and human naive CD4^+^ T cells cultured under iTreg- or Th17-polarizing conditions (right panels). (**C**) Representative Western blot analysis and quantification of TAp63 and Hsp90 in murine naive CD4^+^ T cells from female C57BL/6 mice (8 weeks old) stimulated with plate-bound anti–CD3 mAb and soluble anti–CD28 mAb under indicated conditions for 4 days. Data are reported as mean ± SEM. *n* = 4. *P < 0.05, **P < 0.01 by 1-way ANOVA followed by Tukey’s test. (**D**) Representative Western blot analysis of TAp63 and Hsp90 in murine tTreg cells or iTreg cells. (**E**) Representative Western blot analysis of TAp63 and Hsp90 in human Th17 cells or murine Th17 cells treated with MTX (100 nM) or PBS for 24 hours. (**F**) Representative Western blot analysis of TAp63 and Hsp90 in human Th17 cells or murine Th17 cells treated with NECA (10 μM) or vehicle for 4 days. (**G**) Representative Western blot analysis of TAp63 and Hsp90 in human Th17 cells or murine Th17 cells treated with CSC (10 μM) or vehicle for 4 days and with MTX (100 nM) or PBS for 24 hours.

To search for Th cell subsets that express TAp63, we first analyzed the expression of TAp63 in human CD4^+^ T cell subsets isolated from healthy individuals. As shown in Figure 2A, human Th17 cells expressed higher levels of TAp63 mRNA than did naive CD4^+^ T cells, Th1 cells, and Treg cells. Consistently, the expression of TAp63 proteins was much higher in Th17 cells than in Treg cells or Jurkat cells (Figure 2B). On the other hand, when naive human CD4^+^ T cells were cultured under Th17-polarizing or induced Treg–polarizing (iTreg-polarizing) conditions, not only Th17 cells but also iTreg cells expressed TAp63 (Figure 2B).

We next analyzed the expression of TAp63 proteins in murine Th cell subsets. As shown in Figure 2C, TAp63 was highly expressed in CD4^+^ T cells stimulated under neutral, Th1, Th2, Th17, and iTreg conditions. Consistent with the data of Treg cells isolated from in vivo and iTreg cells in humans (Figure 2B), murine thymic-derived Treg (tTreg) cells, which stably express CD25 and Foxp3 in vivo similarly to human Treg cells, expressed lower levels of TAp63 than murine iTreg cells (Figure 2D). These findings indicate that T cell receptor (TCR) signals induce the expression of TAp63 and that Treg cells developed in vivo expressed lower levels of TAp63 than in vitro–induced Treg cells in humans and mice.

We next examined whether MTX suppresses TAp63 in CD4^+^ T cells in vitro. As shown in Figure 2E, MTX suppressed the expression of TAp63 proteins in Th17 cells in humans and mice. Because it has been reported that MTX increases adenosine levels in extracellular spaces and exerts its immunosuppressive effects via adenosine A2A receptors (7, 23–26), we next analyzed the role of the MTX/adenosine signaling axis in the regulation of TAp63 expression in vitro. Consistent with a previous report showing that the A2A receptor is expressed on effector T cells and A2A receptor agonists suppress their proliferation (27), an adenosine receptor agonist, 5′-*N*-ethylcarboxamidoadenosine (NECA), suppressed the proliferation of Th17 cells (Supplemental Figure 2). However, NECA did not suppress the expression of TAp63 in both human and murine Th17 cells (Figure 2F). Moreover, an A2A receptor antagonist, 8-(3-chloro-styryl)-caffeine (CSC), did not restore the MTX-mediated suppression of TAp63 in both human and murine Th17 cells (Figure 2G). Collectively, TAp63 is expressed in human and murine CD4^+^ T cells, with higher expression levels in Th17 cells in humans, and suppressed by MTX, presumably independent of adenosine signaling.

### Knockdown of TAp63 in SKG CD4^+^ T cells ameliorates arthritis.

To examine the role of TAp63 in CD4^+^ T cells, we constructed murine Tp63-knockdown retrovirus vectors that could target TAp63 but not ΔNp63. Because the 5′ sequence of TAp63 is different from that of ΔNp63, we designed 4 knockdown sequences (mTAp63KD1, mTAp63KD2, mTAp63KD3, and mTAp63KD4) specific for murine TAp63 (Supplemental Figure 3A) and subcloned them into a miRNA-adapted retrovirus vector, LMP-IRES-NGFR. We infected murine CD4^+^ T cells with these knockdown retroviruses and analyzed the expression of TAp63 proteins in the infected NGFR^+^ cells. The knockdown retrovirus of mTAp63KD4 significantly suppressed the expression of endogenous TAp63 in CD4^+^ T cells (Supplemental Figure 3B). Hereafter, we used this vector as a murine TAp63-knockdown (mTAp63KD) retrovirus vector.

**Figure 3:**
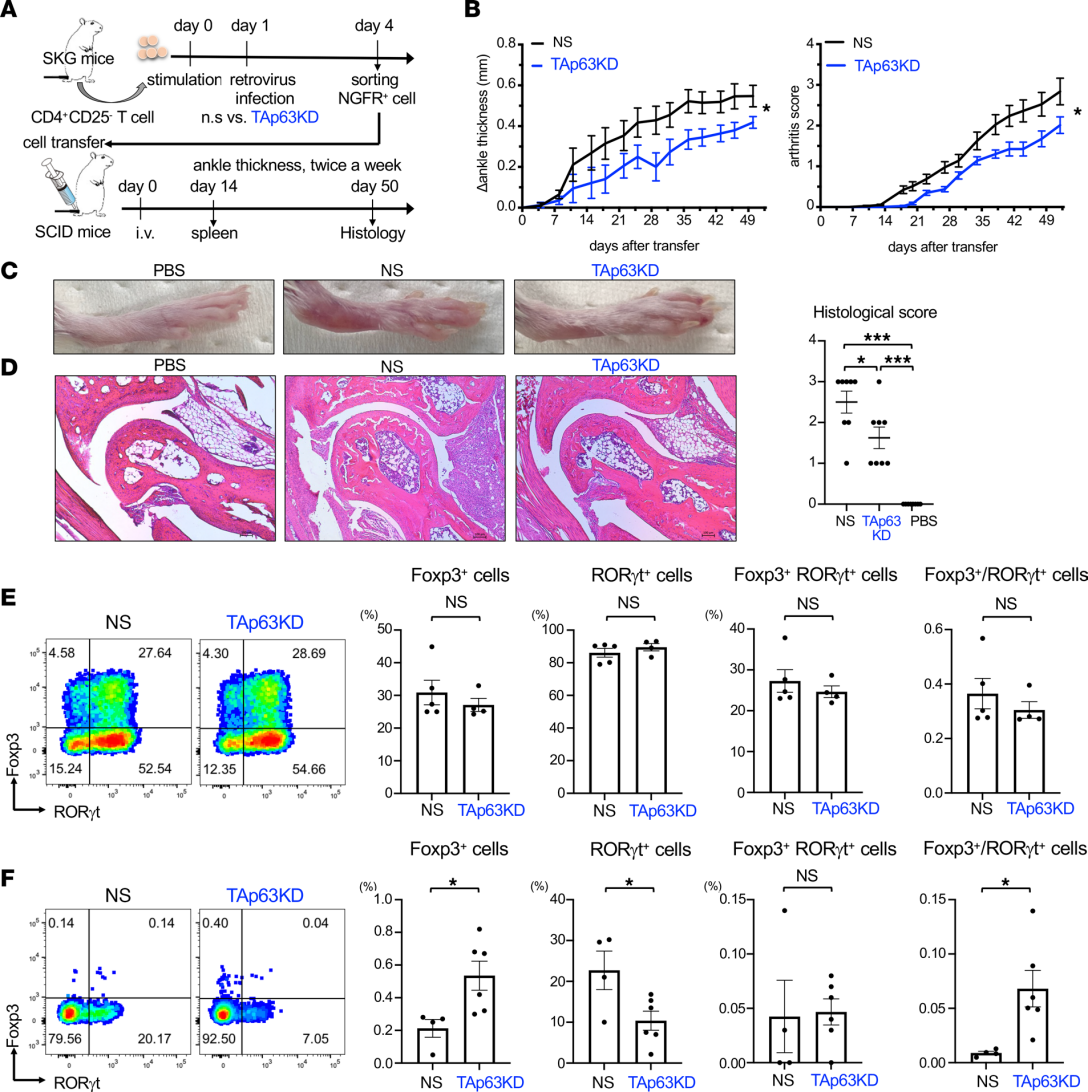
TAp63 knockdown in SKG CD4^+^ T cells ameliorates arthritis. (**A**) CD25^–^CD4^+^ T cells from SKG mice were infected with retroviruses of mTAp63KD or nonsilencing (n.s. or NS) in Th17-polarizing conditions, and infected NGFR^+^ cells were sorted (TAp63KD SKG CD4^+^ T cells or n.s. SKG CD4^+^ T cells, respectively) and injected i.v. into SCID mice. Joint swelling was monitored twice a week for 50 days. (**B**) Changes in ankle thickness (left panel) or arthritis score (right panel) in mice transferred with NS SKG CD4^+^ T cells and TAp63KD SKG CD4^+^ T cells. Each symbol and vertical line represent the mean ± SEM. Data are compiled from 3 independent experiments (*n* = 7–8 each). *P < 0.05 by 2-way ANOVA. (**C** and **D**) Representative photograph of hind paw (**C**) and photomicrographs of ankle joints (**D**) of mice injected with PBS, NS SKG CD4^+^ T cells, or TAp63KD SKG CD4^+^ T cells. Scale bar: 100 μm. ***P < 0.001 by 1-way ANOVA followed by Tukey’s test. Histologic scores for the ankle joints of mice injected with PBS, NS SKG CD4^+^ T cells, or TAp63KD SKG CD4^+^ T cells. (**E**) Representative flow cytometric analyses of RORγt versus Foxp3 and the frequencies of Foxp3^+^ cells, RORγt^+^ cells, and Foxp3^+^RORγt^+^ cells of NGFR^+^CD4^+^ T cells, as well as the ratio of Foxp3^+^ to RORγt^+^ cells (right panel) in NGFR^+^CD4^+^ T cells at the timing of cell transfer. Data are reported as mean ± SEM. *n* = 5 (NS) and *n* = 4 (mTAp63KD). (**F**) Representative flow cytometric analyses of RORγt versus Foxp3 and the frequencies of Foxp3^+^ cells, RORγt^+^ cells, and Foxp3^+^RORγt^+^ cells of NGFR^+^CD4^+^ T cells, as well as the ratio of Foxp3^+^ to RORγt^+^ cells (right panel) in splenic NGFR^+^CD4^+^ T cells 14 days after the cell transfer. Data are reported as mean ± SEM. *n* = 4 (NS) and *n* = 6 (mTAp63KD). *P < 0.05 by unpaired *t* test.

SKG mice, which have a missense mutation in Zap70 resulting in impaired negative selection, spontaneously develop autoimmune arthritis pathologically similar to human RA (28). In addition, CD4^+^ T cells isolated from SKG mice induce arthritis if transferred to immunodeficient SCID mice (29). We used this model to evaluate the role of TAp63 in CD4^+^ T cells in autoimmune arthritis. We isolated CD25^–^CD4^+^ T cells from the spleen and lymph nodes of SKG mice, stimulated these cells under Th17-polarizing conditions, infected these cells with mTAp63KD or nonsilencing retrovirus, sorted NGFR^+^ infected cells, transferred them into SCID mice, and monitored joint swelling of ankles for 50 days after cell transfer (Figure 3A).

We found that ankle thickness and arthritis score were significantly decreased in mice injected with mTAp63KD retrovirus–infected SKG CD4^+^ T cells compared with those with nonsilencing retrovirus–infected SKG CD4^+^ T cells (Figure 3B). In addition, histologic analyses of the ankle joints demonstrated that synovitis was ameliorated in mice injected with mTAp63KD retrovirus–infected SKG CD4^+^ T cells compared with those with nonsilencing retrovirus–infected SKG CD4^+^ T cells (Figure 3, C and D). Although the frequencies of Foxp3^+^ cells, RORγt^+^ cells, and Foxp3^+^RORγt^+^ cells were not significantly different between mTAp63KD retrovirus–infected SKG CD4^+^ T cells and nonsilencing retrovirus–infected SKG CD4^+^ T cells at the time of cell transfer (Figure 3E), the frequency of Foxp3^+^ cells among NGFR^+^CD4^+^ T cells in the spleen was significantly increased; the frequency of RORγt^+^ cells was significantly decreased in mice injected with mTAp63KD retrovirus–infected SKG CD4^+^ T cells after 14 days of cell transfer (Figure 3F). Notably, Foxp3^+^RORγt^+^ cells were rare in the spleen and did not differ between the 2 groups after 14 days of cell transfer (Figure 3F), suggesting that most Foxp3^+^ cells did not have the inflammatory properties after 14 days of cell transfer. These results indicate that the TAp63 is involved in the arthritis-inducing potential of SKG CD4^+^ T cells, possibly by regulating the balance between Th17 cells and Treg cells.

### TAp63 downregulates Foxp3 in human CD4^+^ T cells.

**Figure 4:**
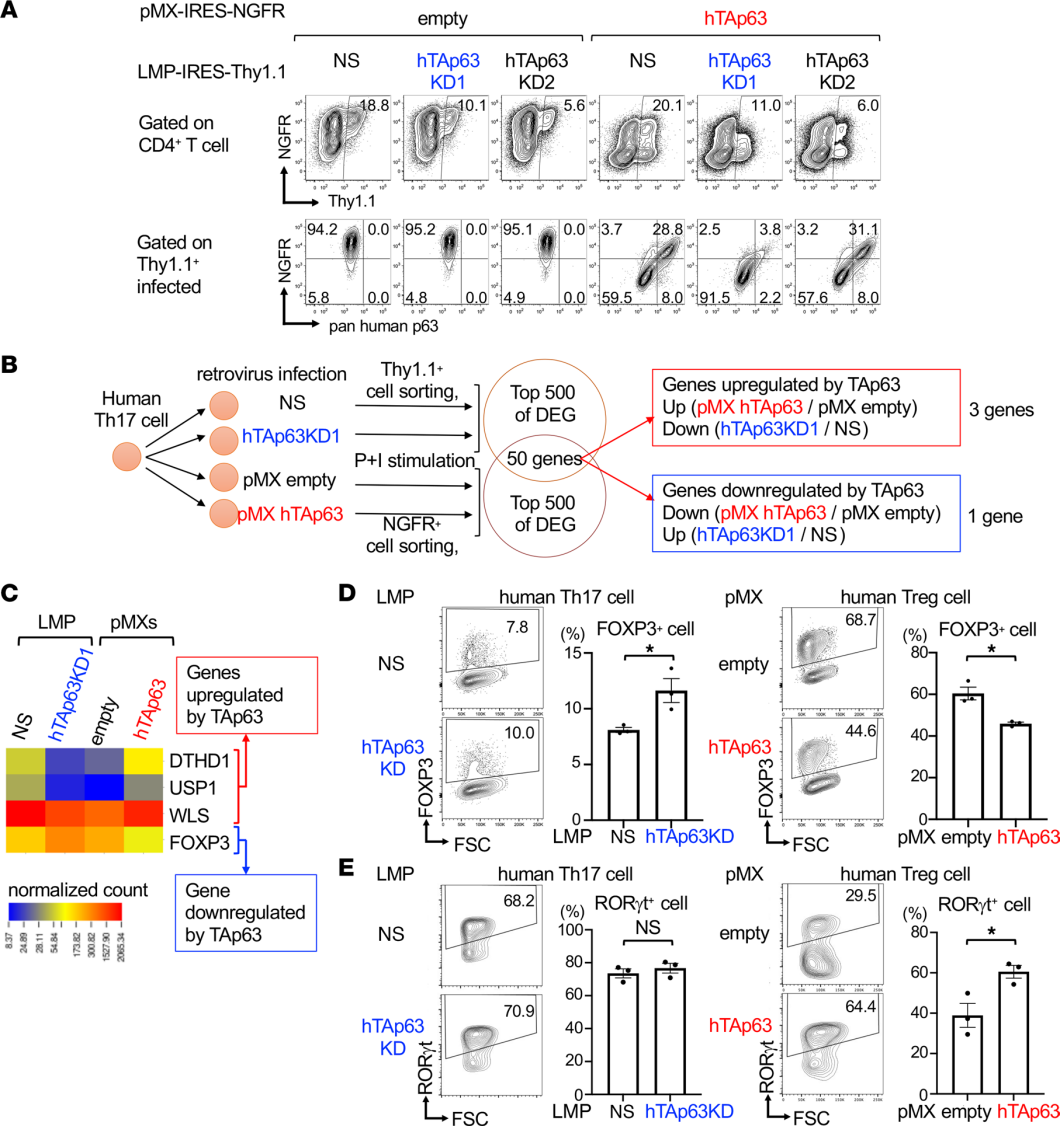
TAp63 downregulates Foxp3. (**A**) Validation of hTAp63-knockdown vectors. CD4^+^ T cells from female C57BL/6 mice (8 weeks old) were infected with retroviruses of pMXs-hTAp63-IRES-NGFR or pMXs-IRES-NGFR empty vector along with retroviruses of LMP-hTAp63KD1-IRES-Thy1.1, LMP-hTAp63KD2-IRES-Thy1.1, or LMP-nonsilencing-IRES-Thy1.1. Representative flow cytometry profiles of the expression of NGFR versus Thy1.1 on CD4^+^ T cells (upper panels) and pan-human p63 versus NGFR on Thy1.1^+^CD4^+^ infected cells (lower panels) are shown. (**B**) Human Th17 cells were infected with retrovirus of either LMP-nonsilencing-IRES-Thy1.1 or LMP-hTAp63KD1-IRES-Thy1.1 and of either pMXs-IRES-NGFR or pMXs-hTAp63-IRES-NGFR. Infected cells were sorted and subjected to RNA-Seq analysis. By comparing between LMP-hTAp63KD1-IRES-Thy1.1 and LMP-nonsilencing-IRES-Thy1.1 and between pMXs-IRES-NGFR and pMXs-hTAp63-IRES-NGFR, the top 500 DEGs were extracted. Among genes shared with 2 comparisons, 3 genes were upregulated and 1 gene was downregulated by TAp63. (**C**) Normalized count data of the genes upregulated or downregulated by TAp63. (**D** and **E**) In the left panels, human Th17 cells were infected with retroviruses of LMP-nonsilencing-IRES-Thy1.1 or LMP-hTAp63KD1-IRES-Thy1.1. In the right panels, human Treg cells were infected with retroviruses of pMXs-IRES-NGFR or pMXs-hTAp63-IRES-NGFR. Representative flow cytometry profiles and the frequency of FOXP3- or RORγt-expressing cells in infected cells are shown. Data are reported as mean ± SEM. *n* = 3 experiments. *P < 0.05 by unpaired *t* test. P+I, PMA and ionomycin.

We next tried to identify TAp63-targeted genes in CD4^+^ T cells. To achieve this purpose, we first established the system for the knockdown of human TAp63. We designed 2 knockdown sequences (hTAp63KD1 and hTAp63KD2) specific for TAp63 (Supplemental Figure 4) and subcloned them into miRNA-adapted retrovirus vector LMP-IRES-Thy1.1. We infected murine CD4^+^ T cells with retrovirus of human TAp63 (pMXs-hTAp63-IRES-NGFR [pMXs TAp63]) along with knockdown retrovirus of LMP-hTAp63KD1-IRES-Thy1.1 (hTAp63KD1), LMP-hTAp63KD2-IRES-Thy1.1 (hTAp63KD2), or LMP-nonsilencing-IRES-Thy1.1 (nonsilencing) to evaluate the efficacy of human TAp63 knockdown. We found that hTAp63KD1, but not hTAp63KD2, suppressed the expression of hTAp63 (Figure 4A).

To identify the genes whose expression is regulated by TAp63 in CD4^+^ T cells, human Th17 cells were isolated from PBMCs and infected with hTAp63KD1 or control retrovirus (nonsilencing). In another set of experiments, sorted human Th17 cells were infected with pMXs TAp63 or a control retrovirus (pMXs empty) (Figure 4B). RNA-Seq analysis was performed for the infected cells isolated by the expression of NGFR or Thy1.1. By comparing pairs of hTAp63KD and nonsilencing and of pMXs TAp63 and pMXs empty, we extracted the top 500 DEGs of each pair and found that 50 genes were shared by the 2 pairs. Among them, death domain–containing 1 (*DTHD1*), ubiquitin specific peptidase 2 (*USP2*), and Wntless, or Wnt ligand secretion mediator (*WLS*) were upregulated and *FOXP3* was downregulated by TAp63 (Figure 4C).

Since it has been reported that MTX increases Foxp3 expression (20), we focused on the relationship between TAp63 and FOXP3. Indeed, the knockdown of TAp63 in human Th17 cells increased FOXP3^+^ cells, whereas the ectopic expression of TAp63 in human Treg cells decreased FOXP3^+^ cells (Figure 4D). We also analyzed RORγt expression in these cells, since TAp63 knockdown in murine Th17 cells reduced RORγt expression (Figure 3F). Although the ectopic expression of TAp63 in human Treg cells increased RORγt^+^ cells, the knockdown of TAp63 in human Th17 cells did not decrease RORγt^+^ cells (Figure 4E). These results suggest that TAp63 principally downregulates FOXP3 expression in human CD4^+^ T cells.

### Knockdown of TAp63 in murine iTreg cells induces hypomethylation of the Treg-specific demethylation region in the Foxp3 locus and enhances the suppressive function.

**Figure 5:**
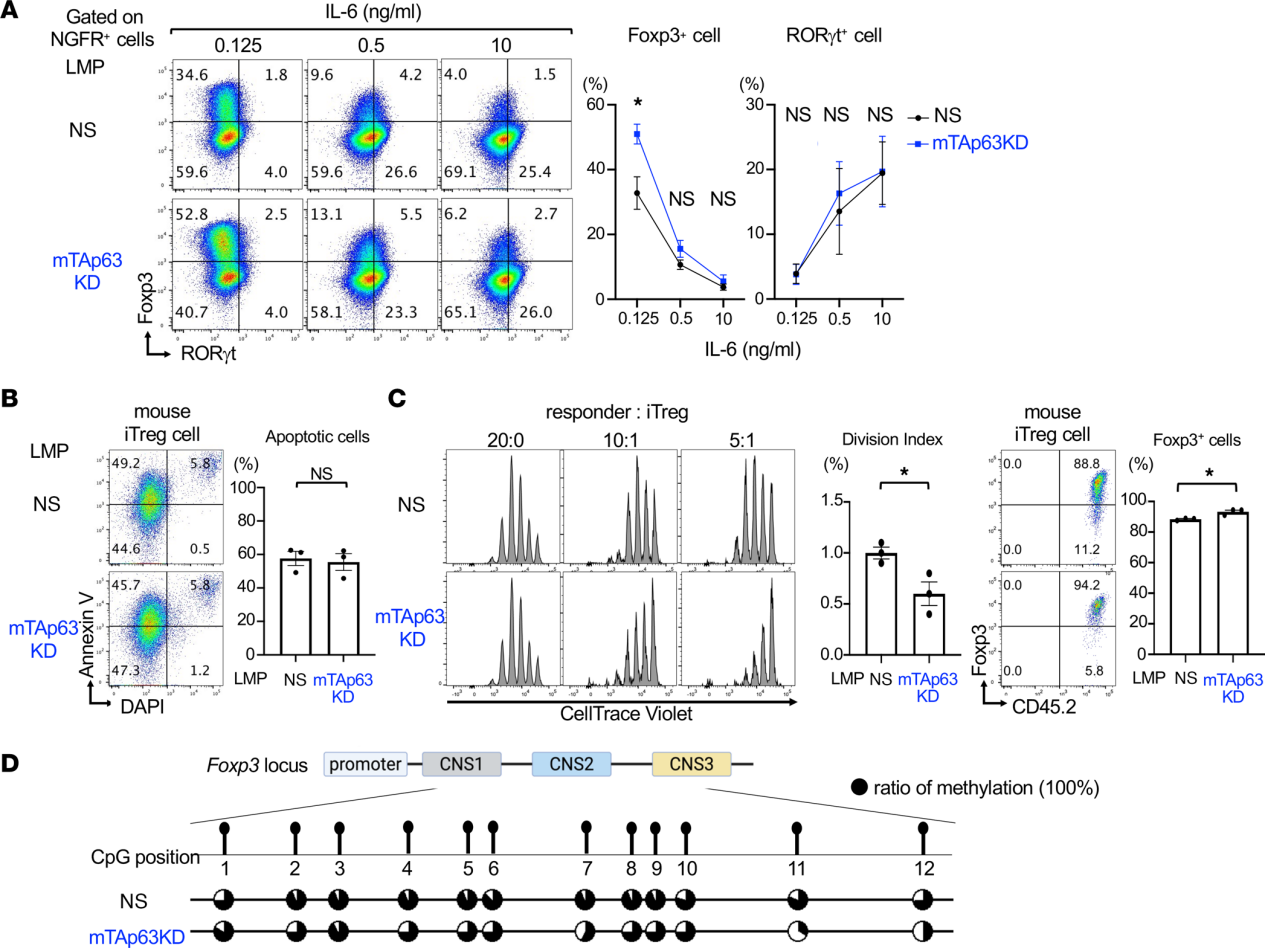
TAp63 knockdown in iTreg cells induces hypomethylation of TSDR in the *Foxp3* locus and enhances the suppressive function. (**A**) Murine naive CD4^+^ T cells were cultured under Th17-polarizing conditions at indicated concentrations of IL-6 and infected with retroviruses of an LMP-IRES-NGFR vector of mTAp63KD or a nonsilencing vector. Representative flow cytometry profiles and the frequency of Foxp3- or RORγt-expressing cells in infected cells are shown. Data are reported as mean ± SEM. *n* = 3–4 experiments. *P < 0.05 by unpaired *t* test. (**B**) Murine naive CD4^+^ T cells were cultured under iTreg-polarizing conditions and infected with retroviruses of LMP-nonsilencing-IRES-NGFR or LMP-mTAp63KD-IRES-NGFR. Representative flow cytometry profiles of annexin V versus DAPI and the frequency of annexin V^+^ cells among DAPI^−^ cells at day 3 are shown (left panels). Data are compiled from 3 independent experiments (*n* = 3 each) and analyzed by unpaired *t* test. (**C**) Naive CD45.2^+^CD4^+^ T cells from Foxp3^YFP-Cre^ mice were cultured under iTreg-polarizing conditions and infected with retroviruses of LMP-mTAp63KD-IRES-NGFR (TAp63KD) or LMP-nonsilencing-IRES-NGFR (NS). Infected NGFR^+^ Foxp3^YFP+^ cells (iTreg) were sorted and co-cultured with CellTrace Violet–labeled naive CD45.1^+^CD4^+^ T cells (responder) at the indicated ratio. Representative histograms of CellTrace Violet and mean ± SEM of division index of responder cells are shown (left panels). Representative flow cytometry profiles of Foxp3 versus CD45.2 and the frequency of Foxp3^+^ cells among CD45.2^+^ cells at day 2 are shown (right panels). Data are compiled from 3 independent experiments (*n* = 3 each). *P < 0.05, unpaired *t* test. (**D**) iTreg cells were prepared as described in **C**, and DNA methylation status for the Foxp3 CNS2 region was determined by the bisulfite sequencing analysis. Data are shown as a lollipop methylation diagram. The black circle indicates a 100% ratio of methylation of CpGs.

We next attempted to address the underlying mechanisms of the Treg-suppressing effect of TAp63. We examined the effect of TAp63 knockdown on the differentiation of murine Th17 and iTreg cells in Th17-polarizing conditions with different concentrations of IL-6. As shown in Figure 5A, TAp63 knockdown increased Foxp3 expression in Th17-polarizing conditions with low levels of IL-6 but not with high levels of IL-6. In contrast, TAp63 knockdown did not affect the expression of RORγt in Th17-polarizing conditions regardless of IL-6 concentrations (Figure 5A).

Because previous studies have shown that TAp63 induces apoptosis in baby hamster kidney cells (30), we first examined the effect of TAp63 on the apoptosis of iTreg cells and found that the knockdown of TAp63 in murine iTreg cells did not show any antiapoptotic effect on iTreg cells (Figure 5B). We next examined whether the knockdown of TAp63 in murine iTreg cells would increase its suppressive function. As shown in Figure 5C, mTAp63KD retrovirus–infected iTreg cells more strongly suppressed the division of responder cells than nonsilencing retrovirus–infected iTreg cells. Consistently, the frequency of Foxp3^+^ cells in mTAp63KD retrovirus–infected iTreg cells was higher than that in nonsilencing retrovirus–infected iTreg cells (Figure 5C).

Treg cell development requires both Foxp3 induction and Treg-specific epigenetic changes (14). Therefore, we examined whether the knockdown of TAp63 affected the methylation status of the conserved noncoding sequence 2 (CNS2) in the Foxp3 locus, where the Treg-specific demethylation region (TSDR) exists. Bisulfite sequencing showed that, consistent with a previous report (13), the TSDR in the Foxp3 locus was strongly methylated in nonsilencing retrovirus–infected iTreg cells. Notably, the knockdown of TAp63 in iTreg cells resulted in hypomethylation of the Foxp3 CNS2 region (Figure 5D). These results suggest that TAp63 contributes to the methylation of the Foxp3 CNS2 region and inhibits the suppressive function of iTreg cells.

### TAp63 suppresses the activation of Foxp3 CNS2 enhancer.

**Figure 6:**
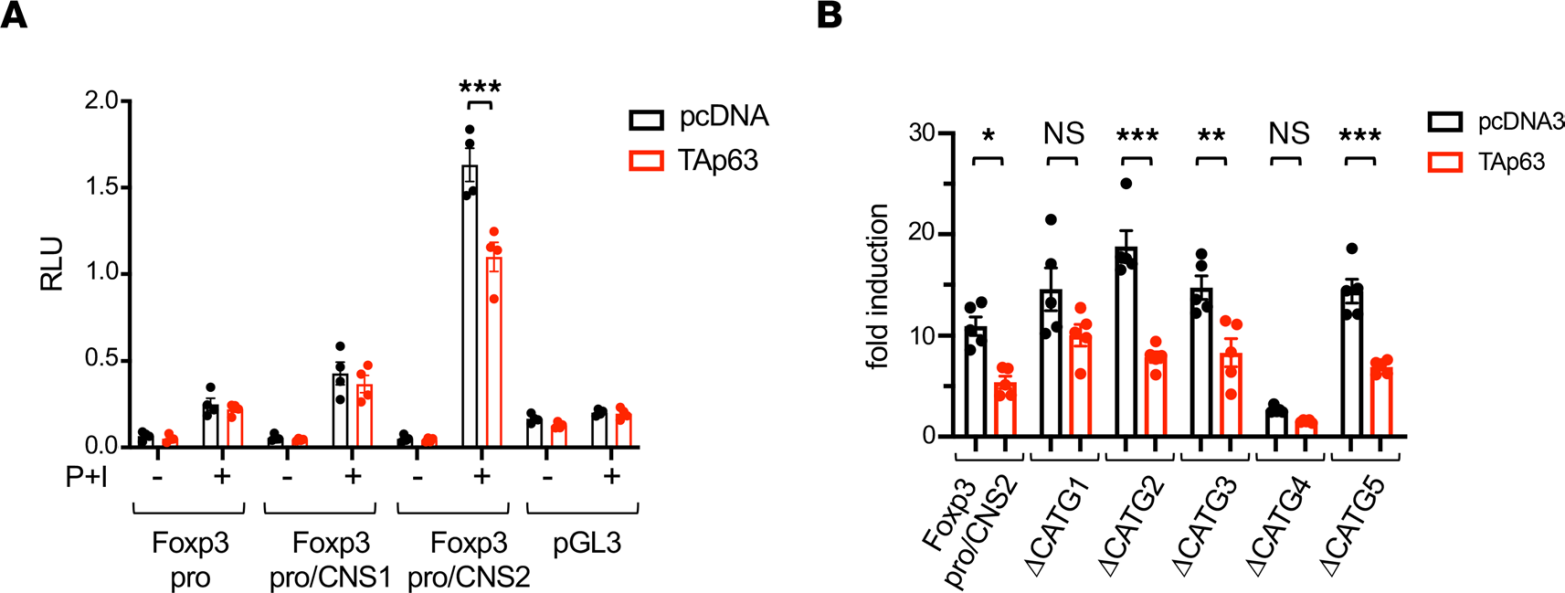
TAp63 suppresses the activation of Foxp3 CNS2 enhancer. (**A**) Jurkat cells were transfected with Foxp3pro, Foxp3pro/CNS1, Foxp3pro/CNS2, or control pGL3 vector along with pcDNA3-TAp63 or empty pcDNA3 vector. Cells were rested for 6 hours and then left unstimulated or stimulated with PMA and ionomycin (P+I) for 12 hours, and luciferase assays were performed. Data are compiled from 4 independent experiments. ***P < 0.001 by 2-way ANOVA followed by Sidak’s test. (**B**) Jurkat cells were transfected with Foxp3pro/CNS2 or its mutant constructs for each putative TAp63 binding site (see Supplemental Figure 5) along with pcDNA3-TAp63 or empty pcDNA3 vector. Cells were stimulated with P+I, and luciferase assays were performed. Data are reported as fold-induction relative to control pGL3-transfected cells. Data are compiled from 5 independent experiments. *P < 0.05, **P < 0.01, ***P < 0.001 by 1-way ANOVA followed by Tukey’s test.

To explore the mechanisms underlying TAp63-mediated suppression of Foxp3 expression, we finally performed reporter assays for the promoter and enhancer of Foxp3. Jurkat cells, which lack the expression of TAp63 protein (Figure 2B), were transfected with several reporter constructs of Foxp3 promoter and enhancer along with a TAp63-expressing vector (pcDNA3-TAp63) or a control empty vector (pcDNA3). As shown in Figure 6A, TAp63 suppressed the activity of Foxp3 promoter plus CNS2 enhancer (Foxp3pro/CNS2), but not Foxp3 promoter alone (Foxp3pro) or Foxp3 promoter plus CNS1 enhancer (Foxp3pro/CNS1), suggesting that CNS2 is a target of TAp63.

Given that TAp63 prefers CATG sequences and there are 5 CATG sequences in the Foxp3 CNS2 enhancer (Supplemental Figure 5), we introduced a mutation to each CATG sequence in the CNS2 enhancer and examined the effect of TAp63. Importantly, TAp63 could not significantly suppress the activation of ΔCATG1 and ΔCATG4 (Figure 6B). Because CATG4 but not CATG1 is conserved between humans and mice and CATG4 is surrounded by Ets-binding and cAMP responsive element–binding protein (CREB-binding) sequences (31, 32) (Supplemental Figure 5), CATG4 seems to be more critical for TAp63 binding than CATG1.

## Discussion

We show that TAp63, an isoform of Tp63, suppresses the activation of the Foxp3 CNS2 enhancer and inhibits Treg cell differentiation. We found that *TP63* was the most significantly downregulated gene in CD4^+^ T cells after MTX treatment in patients with RA (Figure 1A and Table 1). Consistent with the data of patients with RA, TAp63 was highly expressed in human and murine Th17 cells, and MTX inhibited its expression in vitro (Figure 2). Notably, TAp63 was highly expressed in murine and human iTreg cells but not in murine tTreg cells or human Treg cells isolated in vivo (Figure 2). The knockdown of TAp63 in SKG CD4^+^ T cells ameliorated autoimmune arthritis, increased Foxp3^+^ cells, and decreased RORγt^+^ cells in vivo (Figure 3). Comparing the comprehensive gene expression profiles between human Th17 cells overexpressing TAp63 and those with TAp63 knockdown revealed that Foxp3 was one of the genes downregulated by TAp63 (Figure 4). Indeed, TAp63 knockdown in iTreg cells promoted Foxp3 CNS2 hypomethylation, increased Foxp3 expression, and enhanced its suppressive activity (Figures 4 and 5). Reporter assays showed that TAp63 suppressed the activation of the Foxp3 CNS2 enhancer (Figure 6). These findings suggest that TAp63, one of the MTX targets in RA, suppresses Foxp3 expression and exacerbates autoimmune arthritis.

We found that TAp63 was highly expressed in activated Th cells and iTreg cells but not in tTreg cells. Although the processes underlying the low levels of TAp63 in tTreg cells still need to be investigated, the induction of TAp63 in activated murine Th cells occurs during the initial phase of T cell differentiation regardless of cytokine signals and probably through the TCR signaling pathways (Figure 2C). Given that naive CD4^+^ T cells stimulated with TCR and TGF-β upregulate both RORγt and Foxp3 within 48 hours (33), TAp63 may tune Th17 cell differentiation by destabilizing Foxp3 expression during the initial phase of T cell differentiation. We also found that the transfer of TAp63-silenced developing Th17 cells to SCID mice decreased RORγt expression in donor T cells in vivo (Figure 3F). On the other hand, the knockdown of TAp63 in Th17 cells did not affect RORγt expression in vitro (Figure 5A). Since it took us 14 days to analyze the expanded donor T cells after the cell transfer to SCID mice, the discrepancy may be caused by a secondary effect of the expanded Treg cells’ suppression on Th17 cells in the recipient mice.

Meanwhile, we found that the forced expression of human TAp63 induced RORγt expression in human Treg cells, whereas the knockdown of human TAp63 in human Th17 cells did not reduce RORγt expression. In this regard, we have reanalyzed the public data of ChIP-sequencing data with anti-p63 antibody in mammary glands and keratinocytes (34, 35) and found that p63 could bind to the human *RORC* locus (our unpublished data). Therefore, TAp63 may influence the balance between Th17 cells and Treg cells by binding to the *RORC* gene locus and inducing RORγt expression if TAp63 is highly expressed in CD4^+^ T cells. Additional analyses are required to determine whether TAp63 influences the balance between Th17 cells and Treg cells by inducing RORγt under physiological conditions.

We found that higher amounts of IL-6 can overcome the effect of TAp63 knockdown on Foxp3 induction under Th17-polarizing conditions. It has been reported that Treg cell differentiation is markedly inhibited by an excess of IL-6, as in IL-6–transgenic mice (36). It has also been reported that IL-6 induces DNA methyltransferase 1 expression (37), which might inhibit the differentiation into Treg cells by inducing Foxp3 CNS2 methylation (38, 39). These mechanisms may be related to the overcoming of the TAp63-knockdown effect by higher amounts of IL-6 (Figure 5A). However, because TAp63 knockdown induces Treg cell differentiation in in vivo situations (Figure 3), it is likely that TAp63 inhibits Foxp3 induction and Treg cell differentiation under physiological conditions.

We show that TAp63 suppresses Foxp3 CNS2 enhancer activity and maintains methylation of the Foxp3 CNS2 locus in Treg cells. Regarding the induction of hypomethylation of Foxp3 CNS2, it has been reported that Tet family enzymes mediate the hypomethylation and that Cbf-b-Runx1 complex or CREB/ATF is required to maintain the hypomethylation (40). It has also been shown that ΔNp63 associates with Dnmt3a at enhancers and maintains DNA hydroxymethylation to permit the active state in a Tet2-dependent manner in human epidermal stem cells (41), suggesting that ΔNp63 seems crucial for the permission of open chromatin. These findings raised the possibility that ΔNp63 induces open chromatin at the Foxp3 CNS2 enhancer in a Tet-dependent manner, and TAp63 competes with ΔNp63 to maintain DNA methylation. Nevertheless, in our preliminary experiment, ectopic expression of ΔNp63 also suppressed the expression of Foxp3 in iTreg cells. Thus, the possibility mentioned earlier is not likely, and more studies are required to reveal how TAp63 maintains the Foxp3 CNS2 locus methylation in Treg cells.

Regarding the relationship between TP63 and noncancer human diseases, a genome-wide meta-analysis revealed that *TP63* is associated with psoriasis (42). ΔNp63 is a dominant isoform of the skin and is vital for maintaining epidermal stem cells (39). TAp63 is downregulated in psoriatic lesions compared with normal skin (43). Since ΔNp63 binds to DNA and induces open chromatin regions around the genes involved in epidermal fate (44), ΔNp63 may be involved in the pathogenesis of psoriasis by premising open chromatin in epidermal cells. Our findings that the knockdown of TAp63 in CD4^+^ T cells reduces the severity of SKG cell-transfer models of arthritis raises the possibility that an imbalance of TAp63 and ΔNp63 could be involved in the pathogenesis of psoriatic arthritis, which occurs frequently in patients with psoriasis.

We found that *TP63* is the most significantly downregulated gene after MTX treatment in patients with RA and that MTX suppresses TAp63 expression in Th17 cells in vitro. Regarding the mechanisms of MTX action on RA, the efficacy of MTX is believed to be mediated by folate antagonism or increased adenosine signaling through the inhibition of ATIC by methotrexate polyglutamate (7). In the latter case, MTX induces the release of ATP from intracellular to extracellular space, CD39 converts ATP to AMP deaminase, CD73 converts AMP to adenosine, and then adenosine exerts its immunosuppressive effect through A2A receptors (23). It has been reported that Treg cells express high levels of CD39 and CD73 and are involved in this pathway (24–26). However, we found that an adenosine receptor agonist and an A2A receptor antagonist did not significantly affect the expression of TAp63, suggesting that adenosine signaling might not be involved in regulating TAp63 expression. Another pathway associated with the MTX action on T cells may be through the MTX-induced DNA damage. MTX induces long intergenic RNA-p21 via DNA-dependent protein kinase catalytic subunit, a molecular sensor of DNA damage, and suppresses NF-κB activation (15, 16). Nevertheless, since DNA damage generally induces TAp63 in many cell types (45), it is unlikely that MTX-induced DNA damage is the mechanism for MTX-induced TAp63 repression. Collectively, it is plausible that folate antagonism, rather than adenosine signaling or DNA damage, seems to be involved in MTX-mediated suppression of TAp63 in Th17 cells in patients with RA.

This study has several limitations. First, we have not addressed the mechanisms underlying the low expression levels of TAp63 in tTreg cells. We found that tTreg cells expressed TAp63 at a considerably lower level than did iTreg cells, suggesting that the expression of TAp63 is differently regulated in tTreg cells and iTreg cells (Figure 2). Because TAp63α is SUMOylated and degraded by Ubc9, and because Ubc9 is required for the suppressive function of Treg cells (46, 47), it is possible that Ubc9 SUMOylates and degrades TAp63α in tTreg cells. More studies are required to address this possibility.

Second, we have not analyzed the roles of 3 genes (*DTHD1*, *WLS*, and *USP2*) upregulated by TAp63 in CD4^+^ T cells (Figure 4C). DTHD1 is a protein that contains a death domain, but the function of DTHD1 is currently unknown. WLS is a transporter essential for the secretion of Wnt (48), but the function of WLS in T cells is poorly understood. USP2 is a deubiquitinating enzyme that modulates cell cycle progression through the deubiquitination of cyclins and Aurora-A. The role of USP2 in immune and inflammatory signaling is still controversial (49). Thus, additional studies are required to address the roles of DTHD1, WLS, and USP2 in the context of TAp63 in CD4^+^ T cells.

In conclusion, TAp63, a possible target of MTX in CD4^+^ T cells, suppresses Foxp3 expression, exacerbating autoimmune arthritis. These findings should provide new insight into the mechanism of MTX action and the maintenance of Treg cells in autoimmune arthritis.

## Methods

### Patients.

Patients who fulfilled the American College of Rheumatology 1987 revised criteria for the classification of RA and received MTX therapy as a first antirheumatic drug were consecutively recruited at Chiba University Hospital and Asahi General Hospital, as described previously (21). Patients with RA who received treatment with biologic antirheumatic drugs (TNF antagonists, TCZ, or ABT) were also recruited as controls. Patients received routine clinical care and underwent clinical and laboratory assessment, which included 28-joint counts for swelling and tenderness, patient’s and physician’s global assessment of disease activity on a visual analog scale, erythrocyte sedimentation rate (ESR), and serum C-reactive (CRP) protein levels, at baseline and 3 months after the treatment. Patients’ characteristics and disease activity are listed in Table 2 (21).

### DNA microarray analysis.

Peripheral blood samples were obtained at baseline and 3 months after the treatment. PBMCs were isolated using Ficoll-Paque Premium 1.073 (GE Healthcare), as previously described (50), and CD4^+^ T cells were further enriched by using a human CD4^+^ T cell isolation kit (Miltenyi Biotec) (51–53). Total cellular RNA was extracted using Isogen (Nippon Gene). DNA microarray analysis was performed using a Quick Amp labeling kit and a Whole Human Genome DNA Microarray 4×44K according to the manufacturer’s protocol (Agilent). Signal intensity was normalized by adjusting the data to a 75th percentile value. A Linear Models for Microarray Data (Limma) package in R software was used to identify candidate probes (54). We used Limma methods with the thresholds of *q* < 0.05 and *P* < 0.05 to identify the microarray probes whose signal intensity was significantly changed after 3 months of MTX treatment. Table 1 lists microarray probes that matched the criteria, consisting of 18 probes, including 13 probes representing known genes. The signal intensity decreased for all probes except 2 (A_23_P324523 [IQCK] and A_23_P23705 [SPATA6]). The probe whose signal intensity decreased most significantly after MTX treatment was A_23_P327380 (*P* = 4.24 × 10^–9^; *q* = 0.00017) that represents TP63, a member of the p53 family of proteins.

### qPCR analysis.

qPCR was performed as described previously (55). The expression levels of target genes were normalized to those of human GAPDH. DNA sequences of qPCR primers are listed in Supplemental Table 1.

### Mice.

C57BL/6 mice, BALB/c SKG mice, and C.B-17/lcr SCID mice were purchased from CLEA Japan, Inc. C57BL/6 Ly5.1 congenic mice were obtained from RIKEN BRC. C57BL/6 Foxp3^YFP-Cre^ mice were obtained from the Jackson Laboratory. All mice were housed in microisolator cages under specific pathogen–free conditions.

### Reagents.

Antibodies are listed in Supplemental Table 2. Recombinant murine IL-2, human IL-2, and murine IL-6 were purchased from Miltenyi Biotec. Recombinant human IL-1β, IL-23, and TGF-β were purchased from R&D Systems. NECA and CSC were purchased from Merck.

### Cell isolation and cell culture.

Murine CD4^+^ T cells and naive CD4^+^ T cells were isolated using a MojoSort mouse CD4 T cell isolation kit (BioLegend) and a naive CD4^+^ T cell isolation kit (Miltenyi Biotec), respectively. Murine CD4^+^ T cells were stimulated with plate-bound anti–CD3ε mAb (1 μg/mL) in the presence of anti–CD28 mAb (1 μg/mL) (anti–CD3/CD28 mAb) in RPMI medium supplemented with 10% FBS, 2 μM 2-ME, penicillin, and streptomycin (complete RPMI medium). Cells were cultured under neutral conditions (IL-2; 10 ng/mL); Th1-polarizing conditions (IL-2, 10 ng/mL; IL-12, 1 ng/mL; and anti–IL-4 mAb, 10 μg/mL); Th2-polarizing conditions (IL-2, 10 ng/mL; IL-4, 10 ng/mL; and anti–IFN-γ mAb, 10 μg/mL); Th17-polarizing conditions (IL-6, 10 ng/mL; TGF-β, 1 ng/mL; anti–IL-4 mAb, 10 μg/mL; and anti–IFN-γ mAb, 10 μg/mL); or iTreg-polarizing conditions (IL-2, 10 ng/mL; TGF-β, 3 ng/mL; anti–IL-4 mAb, 10 μg/mL; and anti–IFN-γ mAb, 10 μg/mL). For the analysis of TAp63 in tTreg cells and iTreg cells, Foxp3^YFP-Cre+^CD25^+^CD4^+^ T cells (tTreg cells) or Foxp3^YFP-Cre–^CD62L^+^CD44^–^ naive CD4^+^ T cells were sorted from Foxp3^YFP-Cre^ mice, stimulated with Dynabeads mouse T-activator CD3/CD28 (Thermo Fisher Scientific), and cultured under tTreg-expansion conditions (IL-2, 100 ng/mL) or iTreg-polarizing conditions, respectively, according to the manufacturer’s instructions.

For the analysis of human T cells, CD45RA^+^CCR7^+^CD4^+^ T cells, CCR6^–^CXCR3^+^CD4^+^ T cells, CCR6^+^CXCR3^+^CD4^+^ T cells, and CD25^+^CD127^lo^CD4^+^ T cells were sorted from PBMCs as naive CD4^+^ T cells, Th1 cells, Th17 cells, and Treg cells, respectively, by an SH800 cell sorter (Sony). Human CD4^+^ T cells were stimulated with 2 μL of Dynabeads human T-activator CD3/CD28 (hCD3/CD28 Dynabeads) (Thermo Fisher Scientific) in 200 μL of complete RPMI medium. Initially, human Th1 cells were cultured with IL-12 (10 ng/mL), IL-2 (10 ng/mL), and anti–IL-4 mAb (10 μg/mL), and human Th17 cells were cultured with IL-1β (50 ng/mL), IL-23 (50 ng/mL), anti–IL-4 mAb (10 μg/mL), and anti–IFN-γ mAb (10 μg/mL). Human T cells were expanded by adding a complete RPMI medium supplemented with IL-2 (10 ng/mL) for Th1 cells and Th17 cells and IL-2 (100 ng/mL) for Treg cells every 2 to 3 days. For the analysis of the expression of TAp63 in human iTreg cells and Th17 cells polarized from naive CD4^+^ T cells, human naive CD4^+^ T cells were stimulated with hCD3/CD28 Dynabeads and cultured under iTreg conditions (TGF-β, 3 ng/mL; IL-2, 100 ng/mL) or Th17-polarizing conditions (IL-1β, 10 ng/mL; IL-23, 100 ng/mL; IL-6, 20 ng/mL; TGF-β, 1 ng/mL) for 3 days.

### Western blot.

Western blot was performed as described previously (56) by using anti-TAp63 (Poly6189; BioLegend), anti-TAp63 (TAp63.4-1; BioLegend), and anti-Hsp90 (H114; Santa Cruz Biotechnology). Quantification of IB was performed by using Image Lab Software (Bio-Rad). The expression levels of target proteins were normalized to those of Hsp90.

### Retrovirus vectors and retrovirus-mediated gene expression.

A retrovirus vector pMXs-IRES-NGFR was described previously (57). MSCV-LTR-miR30-puro-IRES-GFP (LMP) was obtained from Thermo Fisher (58). GFP cassette of LMP vector was substituted by human NGFR and mouse Thy1.1 to make MSCV-LTR-miR30-puro-IRES-NGFR (LMP-IRES-NGFR) and MSCV-LTR-miR30-puro-IRES-Thy1.1 (LMP-IRES-Thy1.1), respectively (57). To make TAp63KD retrovirus vectors, human and murine TAp63 miRNA-adapted shRNA (shRNAmir) were designed at the RNAi codex website (http://cancan.cshl.edu/cgi-bin/Codex/Codex.cgi) and subcloned into LMP vectors according to manufacturer’s instruction. Oligonucleotide DNA sequences for human TAp63, murine TAp63, and nonsilencing shRNAmir are shown in Supplemental Table 3. Retrovirus-mediated gene induction for murine CD4^+^ T cells was performed as described previously (59). For retrovirus-mediated gene induction of human CD4^+^ T cells, after CD4^+^ T cells were stimulated with hCD3/CD28 Dynabeads for 48 hours, CD4^+^ T cells, together with hCD3/CD28 Dynabeads, were transferred to plates covered with retrovirus-bound RetroNectin (Takara Bio) and centrifuged at 800*g* for 90 minutes at 32°C.

### Arthritis induced by T cell transfer of SKG mice.

CD25^–^CD4^+^ T cells were isolated from the spleen and lymph nodes of 4-week-old SKG mice using a MojoSort mouse CD4 T cell isolation kit and biotin-conjugated anti-CD25 mAb (PC61). Cells were cultured in Th17-polarizing conditions and infected with retroviruses of LMP-nonsilencing-IRES-NGFR or LMP-mTAp63KD4-IRES-NGFR. Three days later, retrovirus-infected NGFR^+^CD4^+^ T cells were sorted and transferred i.v. to 8-week-old SCID mice. Joint swelling of the ankles was monitored twice a week for 50 days after the cell transfer, in a blinded manner. Joint swelling was scored as follows: 0 = no joint swelling, 0.1 = swelling of 1 finger joint, 0.5 = mild swelling of wrist or ankle, and 1.0 = severe swelling of wrist or ankle. Scores for all fingers of the forepaws and hind paws, and for wrists and ankles, were totaled for each mouse (29). Hind paws were collected, fixed in 4% paraformaldehyde phosphate buffer solution, and decalcified in 18.5% EDTA for 14 days. Tissues were then paraffin-embedded, sectioned, and stained with H&E. Images were obtained using an Imager.A1 microscope (Zeiss). Histology was scored in a blinded manner as follows: 0 = none, 1 = mild, 2 = moderate, and 3 = severe.

### Cloning of TAp63 isoform expressed in human Th17 cells.

We cloned TAp63 isoforms expressed in human Th17 cells. Three curated isoforms of TAp63, which have different C-terminal sequences (TAp63α [NM_03722], TAp63β [NM_001114978], and TAp63γ [NM_001114979]), have been reported (Supplemental Figure 6A). Nevertheless, these curated isoforms could not be cloned from human Th17 cells (left panel in Supplemental Figure 6A). Thus, we designed forward primers (X1, X2, X4, and X5) for the predicted isoform of human TAp63 (XM_011513251, XM_011513252, XM_011513253, and XM_005247844, respectively), and reverse primers for TAp63α, TAp63β, and TAp63γ (Supplemental Table 4). Consequently, we found that XM_005247844, the predicted isoform of TAp63α, was expressed in human Th17 cells (right panel of Supplemental Figure 6B). Although we also detected PCR products with primer pairs of X5-TAp63β and X2-TAp63γ, the size of these PCR products was different from the expected size. Thus, it is suggested that the TAp63 isoform expressed in human Th17 cells is a TAp63α isoform (XM_005247844) (Supplemental Figure 6B). We cloned the TAp63α isoform (XM_005247844) and subcloned it into pMXs-IRES-NGFR vector to make pMXs-hTAp63-IRES-NGFR.

### RNA-Seq.

RNA-Seq libraries were prepared using a SureSelect Strand-Specific RNA Library Preparation Kit (Agilent). Sequencing was performed on an Illumina HiSeq1500 using a HiSeq Rapid SBS kit (Illumina) in a 50-base, single-end mode. Count data were calculated with htseq-count. An acronym for the tag count comparison (TCC) package in R software was used to differentially express genes (60) and to normalize count data. Genes that were upregulated by overexpressing hTAp63 and downregulated by TAp63 knockdown were considered genes upregulated by TAp63. Genes downregulated by TAp63 are vice versa.

### Intracellular cytokine and transcription factor staining.

Intracellular cytokine staining was performed as described previously (61). FACS profiles were analyzed by FlowJo software (BD Biosciences).

### Luciferase reporter assay.

Jurkat cells were obtained from RIKEN BRC. Jurkat cells were transfected with pcDNA3 vectors (pcDNA3-TAp63 or empty pcDNA3), pGL3 vectors, and pRL-TK using a Neon transfection system (Thermo Fisher Scientific). After cells were cultured for 6 hours, aliquoted cells were left untreated or treated for another 12 hours with PMA (20 ng/mL, Calbiochem) and ionomycin (1 μg/mL, Calbiochem), and then RLUs were assessed with a dual luciferase assay system (Promega) (55). pGL3 vectors of Foxp3 promoter, Foxp3 promoter plus CNS1 enhancer, and Foxp3 plus CNS2 enhancer were described initially as pFoxp3pro, pFoxp3pro/CNS2, and pFoxp3pro/CNS3, respectively (from W. Leonard, NIH, Bethesda, Maryland, USA) (32).

### Suppression assay.

CD4^+^Foxp3^YFP^ cells from Foxp3^YFP-Cre^ mice were sorted by an SH800 cell sorter. Naive CD45.1^+^CD4^+^ responder T cells were purified from CD45.1 congenic mice and stained with CellTrace Violet according to the manufacturer’s instruction (Thermo Fisher Scientific). The suppression assay was performed as described previously (55).

### Apoptosis assay.

Murine naive CD4^+^ T cells were isolated using a naive CD4^+^ T cell isolation kit. Cells were cultured in iTreg-polarizing conditions and infected with retroviruses of LMP-nonsilencing-IRES-NGFR or LMP-mTAp63KD4-IRES-NGFR. Three days later, dead cells were removed using a MojoSort Dead Cell Removal kit (BioLegend), and iTreg cells were restimulated with plate-bound anti–CD3ε mAb (1 μg/mL) and IL-2 (10 ng/mL). Five hours after the restimulation, iTreg cells were stained with annexin V according to the manufacturer’s instruction (BD Biosciences).

### Bisulfite sequencing.

Bisulfite sequencing was performed as described previously (62). Plasmids from 12 colonies were purified and sequenced. Methylation was analyzed by the QUantification tool for Methylation Analysis (http://quma.cdb.riken.jp/top/quma_main.html).

### Statistics.

Statistical analysis was performed using R software (version 3.2.0) or GraphPad Prism (GraphPad Software). All of the *t* tests in this study are 2 tailed. Data are summarized as mean ± SEM. *P* values less than 0.05 were considered significant. The statistical analysis of the results is described in each figure legend.

### Study approval.

The Ethics Committee of Chiba University approved the study design and procedures (reference no. 872). Written informed consent was obtained from the patients in accordance with the Declaration of Helsinki. The Chiba University Animal Care and Use Committee approved the animal procedures used in this study.

### Data availability.

The accession numbers for the DNA microarray and RNA-Seq data sets reported in this article are NCBI Gene Expression Omnibus GSE176440 (DNA microarray) and GSE181427 (RNA-Seq).

## Author contributions

AS, ST, KI, HN, K Suga, AS, Y Sugawara, TK, Y Sanayama, SF, SIK, AI, KH, K Suzuki, and OO were involved in drafting the article and approved the final version to be published. AS has full access to all data in the study and takes responsibility. ST, AS, KI, and HN contributed to the study conception and design. K Suga, ST, AS, Y Sugawara, TK, Y Sanayama, SF, SIK, AI, KH, K Suzuki, and OO conducted the research. K Suga, AS, ST, and KI conducted the statistical analyses. K Suga, AS, ST, KI, and HN prepared the manuscript.

## Supplementary Material

Supplemental data

## Figures and Tables

**Table 1:**
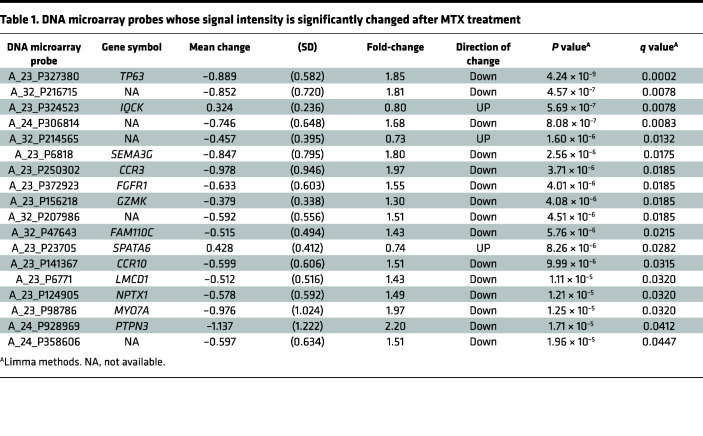
DNA microarray probes whose signal intensity is significantly changed after MTX treatment

**Table 2:**
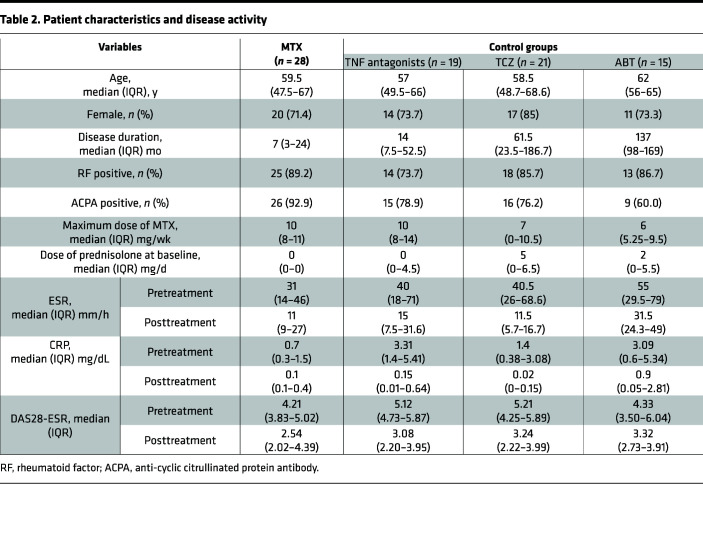
Patient characteristics and disease activity

## Version 1 05/22/2023

Electronic publication
