# Supplementary material for: TAp63, a methotrexate target in CD4+ T cells, suppresses Foxp3 expression and exacerbates autoimmune arthritis
Source: JCI Insight. 2023 May 22;8(10):e164778. doi: 10.1172/jci.insight.164778 (PMC10322677; doi:10.1172/jci.insight.164778)
Supplement: Supplemental data [file jciinsight-8-164778-s097.pdf]

Fig.S1

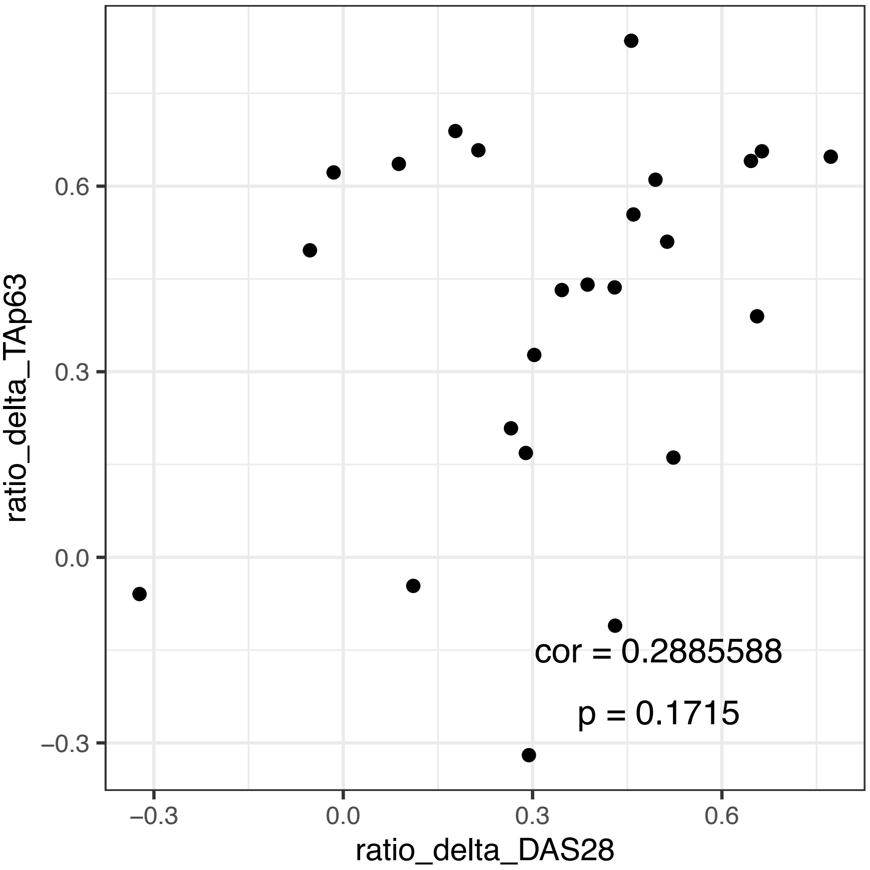

Supplementary Figure 1. Correlation of the change in TAp63 expression and DAS28-ESR

The correlation between the reduction rate of DAS28 ([DAS28 before treatment] – [DAS28 after treatment] / [DAS28 before treatment]) and the reduction rate of TAP63 ([TAp63 signal intensity before treatment] – [TAp63 signal intensity after treatment] / [TAp63 signal intensity before treatment]) was analyzed. Due to the lack of data, 24 subjects were assessed.

Fig.S2

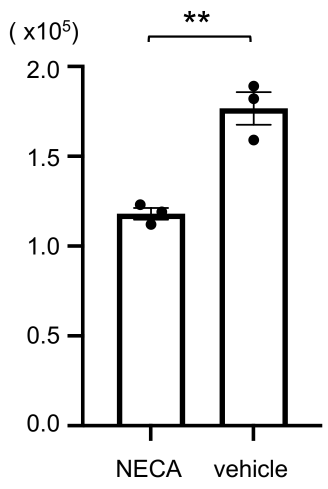

Supplementary Figure 2. NECA suppresses the proliferation of murine Th17 cells
Murine naïve CD4$^+$ T cells were cultured under Th17-polarizing conditions in the presence of NECA (10 μM) or vehicle for 4 days and the number of viable CD4$^+$ T cells was counted. n = 3 (NECA) and n = 3 (vehicle). * * P<0.01 by unpaired t-test.

Fig.S3

A

mTAp63KD1
ATGAATTTTGAAACTTCACGGTGTGCCACCCTACAGTACTGCCCCGACCCTTACATCCAGCGTTTCATAG

mTAp63KD2
AAACCCCAGCTCATTTCTCGTGGAAAGAAAGTTATTACAGATCTGCCATGTCGCAGAGCACCCAGACAAG

CGAGTTCCTCAGCCCAGAGGTCTTCCAGCATATCTGGGATTTTCTGGAACAGCCTATATGCTCAGTACAG

mTAp63KD3
CCCATCGAGTTGAACTTTGTGGATGAACCTTCCGAAAATGGTGCAACAAACAAGATTGAGATTAGCATGG

mTAp63KD4
ATTGTATCCGCATGCAAGACTCAGACCTCAGTGACCCCATGTG

B

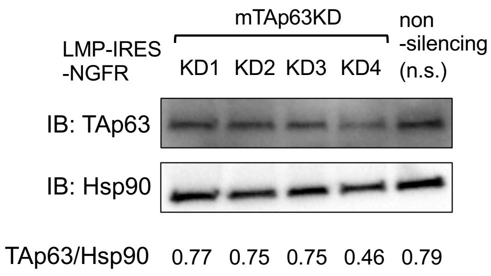

Supplementary Figure 3. Target sequences of murine TAp63 specific shRNAmir
(A) 5' of murine TAp63 coding sequences that are not shared with murine ΔNp63 are shown. Target sequences of murine TAp63 specific shRNAmir are indicated in red. **(B)** Murine naïve $CD4^+$ T cells were cultured under neutral conditions and infected with retroviruses of LMP-IRES-NGFR vectors of mTAp63KD or a non-silencing vector. Infected cells ($NGFR^+$ cells) were sorted and immunoblotted. Representative western blot and quantification analysis of TAp63 and Hsp90 are shown.

Fig.S4

Alignment of coding sequences of human TAp63α and ΔNp63α

```
TAp63alpha_XM_005247844      ---------------------------------------------------ATGCCCAGC 9
TAp63alpha_NM_003722         ATGAATTTTGAAACTTCACGGTGTGCCACCCTACAGTACTGCCCTGACCCTTACATCCAG 60
dNp63alpha_NM_001114980      ------------------------------------------------------------ 0

                                                  hTAp63KD1
TAp63alpha_XM_005247844      TGTTTCGTAGAAACCCCAGCTCATTTCTCTTGGAAAGAAAGTTATTACCGATCCACCATG 69
TAp63alpha_NM_003722         CGTTTCGTAGAAACCCCAGCTCATTTCTCTTGGAAAGAAAGTTATTACCGATCCACCATG 120
dNp63alpha_NM_001114980      ------------------------------------------------------------ 0

TAp63alpha_XM_005247844      TCCCAGAGCACACAGACAAATGAATTCCTCAGTCCAGAGGTTTTCCAGCATATCTGGGAT 129
TAp63alpha_NM_003722         TCCCAGAGCACACAGACAAATGAATTCCTCAGTCCAGAGGTTTTCCAGCATATCTGGGAT 180
dNp63alpha_NM_001114980      ------------------------------------------------------------ 0

TAp63alpha_XM_005247844      TTTCTGGAACAGCCTATATGTTCAGTTCAGCCCATTGACTTGAACTTTGTGGATGAACCA 189
TAp63alpha_NM_003722         TTTCTGGAACAGCCTATATGTTCAGTTCAGCCCATTGACTTGAACTTTGTGGATGAACCA 240
dNp63alpha_NM_001114980      ------------------------------------------------------------ 0

                                                                          hTAp63KD2
TAp63alpha_XM_005247844      TCAGAAGATGGTGCGACAAACAAGATTGAGATTAGCATGGACTGTATCCGCATGCAGGAC 249
TAp63alpha_NM_003722         TCAGAAGATGGTGCGACAAACAAGATTGAGATTAGCATGGACTGTATCCGCATGCAGGAC 300
dNp63alpha_NM_001114980      ------------------------------------------ATGTTGTACCTGGAAAAC 18
                                                                           *   * ** *  **

TAp63alpha_XM_005247844      TCGGACCTGAGTGACCCCATGTGGCCACAGTACACGAACCTGGGGCTCCTGAACAGCATG 309
TAp63alpha_NM_003722         TCGGACCTGAGTGACCCCATGTGGCCACAGTACACGAACCTGGGGCTCCTGAACAGCATG 360
dNp63alpha_NM_001114980      AATGCCCAGACTCAATTTAGTGAGCCACAGTACACGAACCTGGGGCTCCTGAACAGCATG 78
                                * ** ** * *    *    *************************************
```

Supplementary Figure 4. Alignment of coding sequences of human TAp63α and ΔNp63α
Coding sequences of human TAp63α (NM_003722 and XM_005247844) and ΔNp63α (NM_001114980) were aligned by Clustal Omega (https://www.ebi.ac.uk/Tools/msa/clustalo/). Target sequences of shRNAmir were indicated in blue.

Fig.S5

```
                                     kB
m       TGGAGTTTTAAGCTTCCTGGCTTTAGGTGGTTCCCATTTCTTTGGGCTCTGGGACATCAA
h       -----------------------------------GGAGTTTGGACACCAGGGACACTGG
                                                ** *    * ******

                               CATG1
m       TACACACAGTAAGAAGGTGGATCCATGCACCCTACAGAGTCTGTGTTCTTGAGATTCTAA
h       CCTACACATACTGAGACTTTGGGACCGTAGACCCCACAGTCTGTGGTTTTGAGATTCTAG
           *****    **   *        * *  *  ** ******** * ***********

m       AATCCGTTGGCTTTGAGAAATGATATCGTACAGTTCTGAGTTTCTGTTACTACAGCATTT
h       GATCCTTTAAATCTAAGAAATGCTGTTCTATGATTCTGAGGTCCTGGTGTTATACTATTT
         **** **   * * ******* * *  **   ******* * *** *  ** *  ****

                                 CATG2
m       GAAGACTCAAGGGGGTCTCAATATCCATGAGGCCTGCCTAATACTCACCAAGCATCCAAC
h       GAAGACCCCAG-GGGTCCCAGTATCTGTGGAGCCTGCCTGGCACTCTCAGAGCTTCAAAC
        ****** * ** ***** ** ****  **  ********   **** *  *** ** ***

                                                             CATG3
m       CTTGGGCCCCTCTGGCATCCAAGAAAGACAGAATCGATAGA-ACTTGGGTTTTGCATGGT
h       CTGGGTCCTCTCCACAACCCAAGAAGGGCCAGGTCTTCAGAGCTAGGGGCTTGTCATAGT
        ** ** ** ***    * ******* * *    **   ***     *** **  *** **

                                  ETS                            kB
m       AGCCAGATGGACGTCACCTACCACATCCGCTAGCACCCACATCACCCTACCTGGGCCTAT
h       GGCCAGATGGACATCACCTACCACATCCACCAGCACCCATGTCACCCCACCTGGGCCAAG
         *********** *************** * ********  ****** ********* *

                                   GAS
m       CCGGCTACAGGATAGACTAGCCACTTCTCGGAACGAAACCTGTGGGGTAGATTATCTGCC
h       CCTGCTGCAGGACAGGGCAGCCAGTTCTCGGAACGAAACCTGTGGGGTGGGGTATCTGCC
        ** *** ***** **   ***** ************************ *  ********

                                               ETS     CATG4 CREB
m       CCCTTCTCTTCCTCCTTGTTGCCGATGAAGCCCAATGCATCCGGCCGCCATGACGTCAAT
h       CTCTTCTCTTCCTCCGTGGTGTCGATGAAGCCCGGCGCATCCGGCCGCCATGACGTCAAT
        * ************* ** ** ***********   ************************

m       GGCAGAAAAATCTGGCCAAGTTC-AGGTTGTGACAACAGGGCCCAGATGTAGACCCCGAT
h       GGCGGAAAAATCTGGGCAAGTCGGGGGCTGTGACAACAGGGCCCAGATGCAGACCCCGAT
        *** *********** *****    **  ********************* **********

                                                       GAS
m       AGGAAAACATATTCTATGTCCCAGAAACAACCTCCATACAGCTTCTAAGAAACA--GTCA
h       ATGAAAACATAATCTGTGTCCCAGAAACATCCCCCATTCAGCTTCTGAGAAACCCAGTCA
        * ********* *** ************* ** **** ******** ******   ****

                kB
m       AACAGGAACGCCCCAACAGACAGTGCAGGAAGCTGGCTGGCCAGCCCAGCCCTCCAGGTC
h       GAAAGGGACGTCCCAACAGACAGTGCAGGAAGCCGGCTGCCCAGCCCGGCCCTCTAGGTC
         * *** *** ********************** ***** ******* ****** *****

m       CCTAGTACCACTAGACAGACCATATCCAATTCAGGTCCTCTTTCTGAGAATGTACTGATG
h       CTCTACCCCCAGAC--AGATCATCTCCAT------GTCCCTGTCTGAGAATGTATCTATG
        *      **   *   *** ****         * **  ************   ***

m       CATCACACAGTCACACCAGTTCCACAAGTATTTAAGGAGGAGATTTCTTATAAGTTCTGA
h       CTTTGCTGAGTCAGGCCATCCC-ACATGTGTTTGGGGA------GAATTCTTAGCTCTGG
        * *  *  *****  ***   * *** ** ***  ***         ** * ** ****
                                     CATG5
m       CCAAACATAAAGAGCACTTCAAAAGTGACCATGGTCCAGCCATA----------------
h       CCAAGTGTCCAGGCAGCTTCAGAAGTGACCACAGGCCAGCCACATGGGCCAGGCCAGAGT
        ****   *  **    ***** *********  * ******* *
```

Supplementary Figure 5. Putative TAp63 binding sequences in murine and human Foxp3 CNS2

Murine (m) and human (h) Foxp3 CNS2 were aligned by Clustal Omega. Potential STAT (GAS), NF-κB (kB), CREB, and Ets-1–binding sites are boxed in blue. Putative TAp63 binding sequences (CATG1 to CATG5) are indicated in red.

Fig.S6

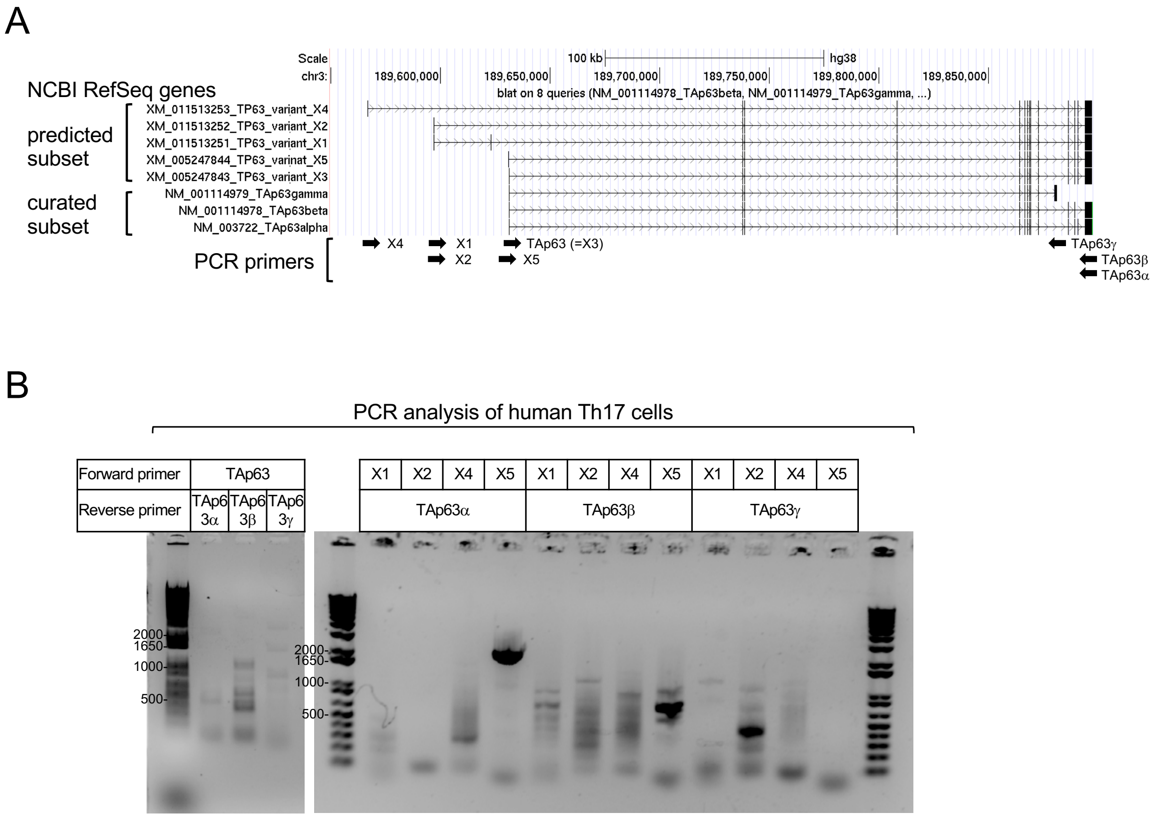

Supplementary Figure 6. Cloning of TAp63a expressed in human Th17 cells
(A) NCBI Reference sequence gene of TP63 (annotation release on 2016) on human Dec. 2013 (GRCh38/hg38) Assembly. PCR primers for the coding sequence of each isoform are shown. DNA sequences of PCR primers are shown in Table 6. **(B)** cDNA from human Th17 cells were PCR amplified with indicated primers and ran on 1% agarose gel.

Supplementary Table 1. qPCR primer sequences

Primer name		Sequences
Human TAp63	forward	5’-TGTATCCGCATGCAGGACT-3’
	reverse	5’-CTGTGTTATAGGGACTGGTGGAC-3’
Human ΔNp63	forward	5’-GAAAACAATGCCCAGACTCAA-3’
	reverse	5’-TGCGCGTGGTCTGTGTTA-3’
Human GAPDH	forward	5’- GAAGGTGAAGGTCGGAGT-3’
	reverse	5’- GAAGATGGTGATGGGATTTC-3

Supplementary Table 2. Antibodies used in this study

Antibodies	Reactivity	Clone	Vendor
CD4	human	S3.5	Thermo Fisher
CD25	human	2A3	BD
CD45RA	human	HI100	BioLegend
CD127	human	A019D5	BioLegend
CCR6	human	G034E3	BioLegend
CCR7	human	G043H7	BioLegend
CXCR3	human	G025H7	BioLegend
NGFR	human	C40-1457	BD
IL-4	human	8D4-8	BioLegend
IFN-γ	human	B27	BioLegend
RORγ(t)	human and mouse	AFKJS-9	Thermo Fisher
Foxp3	human	236A/E7	Thermo Fisher
TAp63	human and mouse	Poly6189	BioLegend
TAp63	Human and mouse	TAp63.4-1	BioLegend
p63	human and mouse	EPR5701	Abcam
rabbit IgG	rabbit	EPR25A	Abcam
CD3ε	mouse	145-2C11	BD
CD4	mouse	RM4-5	BD
CD25	mouse	PC61	BD
CD28	mouse	37.51	BD
CD45.1	mouse	A20	BioLegend
CD45.2	mouse	104	BioLegend
CD62L	mouse	MEL-14	BD
Thy1.1	mouse	OX-7	BD
IL-4	mouse	11B11	BioLegend
IFN-γ	mouse	XMG1.2	BioLegend
Foxp3	mouse	FJK-16s	Thermo Fisher
RORγt	mouse	Q31-378	BD
GFP	Tag	Polyclonal (A21311)	Thermo Fisher

Supplementary Table 3. Oligonucleotide sequences for shRNAmir

name	shRNAmir sequence
Human TAp63 KD1	TGCTGTTGACAGTGAGCGACCAGCTCATTTCTCTTGGAAATAGTGAAGCCACAGATGTATTTCCAAGAGAAATGAGCTGGGTGCCTACTGCCTCGGA
Human TAp63 KD2	TGCTGTTGACAGTGAGCGAATGGACTGTATCCGCATGCAGTAGTGAAGCCACAGATGTACTGCATGCGGATACAGTCCATGTGCCTACTGCCTCGGA
Mouse TAp63 KD1	TGCTGTTGACAGTGAGCGACCTTACATCCAGCGTTTCATATAGTGAAGCCACAGATGTATATGAAACGCTGGATGTAAGGTGCCTACTGCCTCGGA
Mouse TAp63 KD2	TGCTGTTGACAGTGAGCGATCATTTCTCGTGGAAAGAAAGTAGTGAAGCCACAGATGTACTTTCTTTCCACGAGAAATGAGTGCCTACTGCCTCGGA
Mouse TAp63 KD3	TGCTGTTGACAGTGAGCGAGAGTTGAACTTTGTGGATGAATAGTGAAGCCACAGATGTATTCATCCACAAAGTTCAACTCGTGCCTACTGCCTCGGA
Mouse TAp63 KD4	TGCTGTTGACAGTGAGCGAATGGATTGTATCCGCATGCAATAGTGAAGCCACAGATGTATTGCATGCGGATACAATCCATGTGCCTACTGCCTCGGA
non-silencing	TGCTGTTGACAGTGAGCGATCTCGCTTGGGCGAGAGTAAGTAGTGAAGCCACAGATGTACTTACTCTCGCCCAAGCGAGATTGCCTACTGCCTCGGA

Supplementary Table 4. PCR primer sequences for the cloning of human TAp63

PCR primer	DNA sequences
X1	ggatccattATGTCTGAAAGAGAGGTTCAGCAAC
X2	ggatccattATGAAGTGCTGGGAACAGAGAG
X3	ggatccattATGAATTTTGAAACTTCACGGTGTG
X4	ggatccattATGATGGGCCAACAGGCAGAC
X5	ggatccattATGCCCAGCTGTTTCGTAGAAAC
TAp63α	ctcgagTCACTCCCCCTCCTCTTTGATG
TAp63β	ctcgagTCAGACTTGCCAGATCCTGAC
TAp63γ	ctcgagCTATGGGTACACTGATCGGTTTG

BamH1 or Xho1 restriction enzyme sequences were inserted into 5’ sequences of forward primers or reverse primers, respectively. Inserted sequences were shown in lower case.
